# Eosinophil as a cellular target of the ocular anti-allergic action of mapracorat, a novel selective glucocorticoid receptor agonist

**Published:** 2011-12-14

**Authors:** Monica Baiula, Antonino Spartà, Andrea Bedini, Gioia Carbonari, Claudio Bucolo, Keith W. Ward, Jin-Zhong Zhang, Paolo Govoni, Santi Spampinato

**Affiliations:** 1 Department of Pharmacology, University of Bologna, Bologna, Italy; 2 Department of Clinical and Molecular Medicine, University of Catania, Catania, Italy; 3 Pharmaceutical Research & Development, Bausch & Lomb, Rochester, NY; 4 Department of Experimental Medicine - Section of Histology, University of Parma, Parma, Italy

## Abstract

**Purpose:**

Glucocorticoids can either suppress gene transcription (transrepression) or activate it (transactivation). This latter process may contribute to certain side effects caused by these agents. Mapracorat (also known as BOL-303242-X or ZK 245186) is a novel selective glucocorticoid receptor agonist that maintains a beneficial anti-inflammatory activity but seems to be less effective in transactivation, resulting in a lower potential for side effects; it has been proposed for the topical treatment of inflammatory skin disorders. This study assessed the anti-allergic activity of mapracorat at the ocular level and whether eosinophils and mast cells are targets of its action.

**Methods:**

With in vitro studies apoptosis was evaluated in human eosinophils by flow cytometry and western blot of caspase-3 fragments. Eosinophil migration toward platelet-activating factor was evaluated by transwell assays. Interleukin (IL)-6, IL-8, tumor necrosis factor-α (TNF-α), and the chemokine (C-C motif) ligand 5 (CCL5)/regulated upon activation normal T cell expressed, and presumably secreted (RANTES) were measured using a high-throughput multiplex luminex technology. Annexin I and the chemochine receptor C-X-C chemokine receptor 4 (CXCR4) were detected by flow cytometry. With in vivo studies, allergic conjunctivitis was induced in guinea pigs sensitized to ovalbumin by an ocular allergen challenge and evaluated by a clinical score. Conjunctival eosinophils were determined by microscopy or eosinophil peroxidase assay.

**Results:**

In cultured human eosinophils, mapracorat showed the same potency as dexamethasone but displayed higher efficacy in increasing spontaneous apoptosis and in counteracting cytokine-sustained eosinophil survival. These effects were prevented by the glucocorticoid receptor antagonist mifepristone. Mapracorat inhibited eosinophil migration and IL-8 release from eosinophils or the release of IL-6, IL-8, CCL5/RANTES, and TNF-α from a human mast cell line with equal potency as dexamethasone, whereas it was clearly less potent than this glucocorticoid in inducing annexin I and CXCR4 expression on the human eosinophil surface; this was taken as a possible sign of glucocorticoid-dependent transactivation. In the guinea pig, mapracorat or dexamethasone eye drops induced an analogous reduction in clinical symptoms of allergic conjunctivitis and conjunctival eosinophil accumulation.

**Conclusions:**

Mapracorat appears to be a promising candidate for the topical treatment of allergic eye disorders. It maintains an anti-allergic profile similar to that of dexamethasone but seems to have fewer transactivation effects in comparison to this classical glucocorticoid. Some of its cellular targets may contribute to eosinophil apoptosis and/or to preventing their recruitment and activation and to inhibiting the release of cytokines and chemokines.

## Introduction

Allergic eye diseases are usually associated with type 1 hypersensitivity reactions, which cause early and late-phase responses. Clinical symptoms and signs, such as itching, chemosis, and congestion, driven primarily by mast cell degranulation, are manifested very quickly. This is followed by the late-phase response after 6–24 h, which involves eosinophil and neutrophil infiltration into the conjunctiva [1]. Inflammatory cells, cytokines, and proteases contribute to more serious chronic forms [2].

Glucocorticoids are among the most effective drugs for the treatment of allergic eye disease [3]. Their efficacy lies, among other things, in the direct induction of eosinophil apoptosis, suppression of the synthesis and release of eosinophil survival factors, and stimulation of their engulfment by phagocytic cells [4]. Unfortunately, their anti-inflammatory and immunosuppressive effects are frequently accompanied by undesired side effects that may limit their use [5]. At the ocular level, classical glucocorticoids may cause elevation of intraocular pressure and cataract formation [6]. There is, therefore, a pressing need for compounds with the anti-inflammatory potency of standard glucocorticoids but fewer or less troublesome side effects.

The most widely investigated effects of glucocorticoids on target cells involve the regulation of transcription of steroid-responsive genes as a consequence of their penetrating the cytoplasm and binding to the glucocorticoid receptor; then the glucocorticoid–glucocorticoid receptor complex reaches the nucleus and acts as a transcription factor binding to specific DNA sites in the nucleus. This can have two effects on gene transcription: it can either activate transcription (transactivation) by directly binding to the promoter region of target genes or by interacting with other transcription factors, such as activator protein-1 (AP-1), nuclear factor κB (NF-κB), and others, it can suppress transcription (transrepression) [7]. The latter process is considered the key mechanism for the anti-inflammatory activity [8,9]. However, there is also evidence that glucocorticoid-mediated repression of inflammatory genes involves significant post-transcriptional and/or translational mechanisms [10], and the requirement for de novo protein synthesis in glucocorticoid-dependent repression has been highlighted [11]. In contrast, certain side effects are thought to be mediated mainly through transactivation [12].

A better understanding of the molecular mode of glucocorticoid action has led to the identification of novel selective glucocorticoid receptor agonists that should preserve the beneficial anti-inflammatory activity but offer a better side-effect profile [13]. However, the utility of dissociated glucocorticoid ligands as more effective anti-inflammatory compounds with fewer side effects is still debated [11,14], and studies aimed to investigate their pharmacological profile are needed. In fact, so far the majority of these compounds did not enter clinical development.

Recently, Schäcke et al. [15] reported the pharmacological characterization of mapracorat (also known as BOL-303242-X or ZK 245186), a nonsteroidal selective glucocorticoid receptor agonist, for the topical treatment of inflammatory skin disorders. Mapracorat binds with high affinity and selectivity to the human glucocorticoid receptor, possesses potent anti-inflammatory activity, but seems to be less effective in transactivation, resulting in a lower potential for side effects. Mapracorat topically administered as eye drops displays a reduced ability to increase intraocular pressure in normotensive rabbits when compared to dexamethasone [16] and behaves as a partial glucocorticoid receptor agonist in inducing a moderate elevation of myocilin expression in monkey trabecular meshwork cells [17]. Higher levels of myocilin have been related to glucocorticoid-induced ocular hypertension and open-angle glaucoma [17]; however, a putative association between myocilin expression and open-angle glaucoma is still controversial [18]. Conversely, mapracorat and dexamethasone were equally potent in blocking inflammatory cytokine release from cultured human ocular cells [9] and modulating the mitogen-activated protein kinases and nuclear factor kB (NF-kB) signaling cascades [19].

To date, the potential anti-allergic activity of mapracorat in the eye and whether eosinophils and mast cells are targets of its action have had minimal investigation. This study specifically addressed these questions. Adopting in vitro and in vivo models, we found that this novel compound appears to behave as a “differential” glucocorticoid receptor agonist. It maintains an anti-allergic profile similar to that of dexamethasone but seems to have fewer transactivation effects in comparison to this classical glucocorticoid. Schäcke et al. [15] reported that mapracorat, unlike classical glucocorticoids, does not induce apoptosis in a murine thymocyte cell line; in contrast as regards human eosinophils, we have ascertained that it displays higher efficacy than dexamethasone in increasing spontaneous eosinophil apoptosis—an effect related to its anti-allergic activity observed in vivo.

## Methods

### Reagents

(R)-1,1,1-Trifluoro-4-(5-fluoro-2,3-dihydrobenzofuran-7-yl)-4-methyl-2-{[(2-methyl-5-quinolyl)amino]methyl}pentan-2-ol (mapracorat; molecular weight 462.48) was provided by Bausch & Lomb (Rochester, NY), dexamethasone 21-phosphate disodium salt (dexamethasone) was obtained from Sigma-Aldrich (Steinheim, Germany), and mifepristone was purchased from Tocris Bioscience (Bristol, UK). For in vitro studies, mapracorat, dexamethasone, and mifepristone were dissolved in ethanol (10^−2^ M) and further diluted as necessary in cell culture medium. The vehicle was cell culture medium containing 10 μl/ml of ethanol. For in vivo studies, mapracorat eye drops were provided by Bausch & Lomb and further diluted in phosphate buffered saline (PBS; 137 mM NaCl, 2.7 mM KCl, 10 mM Na_2_HPO_4_ x 2 H_2_O, 1.76 mM KH_2_PO_4_, pH 7.4); dexamethasone was dissolved in PBS.

Roswell Park Memorial Institute-1640 (RPMI-1640) plus L-glutamine, penicillin, streptomycin, Alexa Fluor^®^ 488 and 568 conjugated secondary antibody, Hank’s balanced salt solution (HBSS), and MagicMark^TM^ XP Western Standard were purchased from Invitrogen (Carlsbad, CA). PBS, Iscove’s modified Dulbecco’s medium, and heat inactivated fetal bovine serum (FBS) were purchased from Lonza Group Ltd. (Basel, Switzerland). Recombinant human granulocyte-macrophage colony-stimulating factor (GM-CSF) was obtained from R&D Systems (Minneapolis, MN). Interleukin-5 (IL-5), mouse monoclonal anti-chemokine (C-X-C motif) receptor 4 (CXCR4) antibody, mouse IgG_1_ (isotype control), ionomycin from *Streptomyces conglobatus*, anti-β-actin antibody, BSA (BSA), ovalbumin (OVA) grade V, aluminum hydroxide gel, o-phenylenediamine, 30% hydrogen peroxide, Triton X-100, and peroxidase acidic isoenzyme from horseradish were obtained from Sigma-Aldrich. Nuclear and Cytoplasmatic Extraction Reagents kit, bicinchoninic acid (BCA) protein assay, and SuperSignal West Pico chemiluminescent substrate were bought from Pierce (Rockford, IL). Protran^TM^ was obtained from Whatman^®^ (Kent, UK). Anti-caspase-3 antibody was purchased from Cell Signaling (Danvers, MA). Mouse monoclonal anti-annexin I and peroxidase-conjugated secondary antibodies were obtained from Santa Cruz Biotechnology (Santa Cruz, CA). Annexin V-Fluos was obtained from Roche Applied Science (Monza, Italy). Platelet-activating factor (PAF; 1-*O*-Octadecyl-2–0-acetyl-sn-glycero-e-phosphoryl choline) was purchased from Sigma-Aldrich and dissolved in ethanol (2 mM) and further diluted in RPMI 1640 containing 0.1% BSA. Polyacrylamide gel, N,N,N',N'-tetramethylethylenediamine, ammonium persulfate hematoxylin-Bierbrich scarlet solution, lithium carbonate, and sodium dodecyl sulfate were purchased from Sigma-Aldrich. All other reagents were of analytical grade or the highest purity available, purchased from Sigma-Aldrich. All plastic disposables were from Sarstedt (Verona, Italy).

### Cell culture

Human eosinophils, isolated from whole blood by density centrifugation followed by negative selection using immunomagnetic anti-CD16 beads (purity and viability were >95%), were purchased from 3H Biomedical AB (Uppsala, Sweden) and routinely cultured in RPMI 1640 supplemented with 10% FBS, antibiotics (100 U/ml penicillin and 100 μg/ml streptomycin), GM-CSF (70 pM), and IL-5 (30 pM). Before each experiment, cells were maintained in RPMI 1640 medium containing 0.1% FBS and in the absence of GM-CSF and IL-5.

A human mast cell line (HMC-1) [20], obtained from Pio Conti (University of Chieti, Chieti, Italy), was grown in Iscove’s modified Dulbecco’s medium containing 10% FBS, 100 U/ml penicillin, and 100 μg/ml streptomycin in a humidified atmosphere with 5% CO_2_ in air at 37 °C.

### Animals

Male Dunkin-Hartley guinea pigs (250–300 g) were purchased from Charles River (Calco, Italy). Animal procedures were performed in accordance with the Declaration of Helsinki, followed the guidelines of the University of Bologna Animal Care and Use Committee, and were comparable to those published by the Institute for Laboratory Animal Research.

### Eosinophil cell apoptosis

To assess glucocorticoid-induced apoptosis, cells were double stained with annexin V-Fluos and propidium iodide (PI). Annexin V-Fluos was used according to the manufacturer’s instructions. Briefly, the cells were washed in PBS and suspended in annexin V-Fluos labeling solution (10 mM Hepes/NaOH, pH 7.4, 140 mM NaCl, 5 mM CaCl_2_) with PI added (1 µg/ml). The suspension was incubated at room temperature for 10 min and analyzed in the BD FACS Canto II flow cytometry system (Becton, Dickinson and Company, Franklin Lakes, NJ). Eosinophils were gated on the basis of their forward and side light scatter, with cell debris excluded from analysis. Cells showing positive staining with annexin V (i.e., both early apoptotic annexin V^+^/PI^-^ cells and late apoptotic/secondary necrotic cells annexin V^+^/PI^+^) were considered to be apoptotic. A two-way dot plot was prepared to verify the percentage of apoptotic cells. Annexin V^-^/PI^-^ cells were used as control, and annexin V^-^/PI^+^ cells were considered necrotic [21].

### Flow cytometry

Fluorescence-activated cell sorting (FACS) was performed to measure cell surface expression of annexin I and CXCR4 as indicators of glucocorticoid-mediated transactivation [22]. Human eosinophils were double-stained with a red dye-conjugated secondary antibody to trace changes in the expression of CXCR4 or annexin I and a green dye-conjugated annexin V to exclude apoptotic cells from the analysis. The cells were counted and transferred to a 24-well plate (10^6^ cells/well) and serum starved (0.1% FBS) for 24 h. They were then exposed to dexamethasone or mapracorat for 24 h at 37 °C in 5% CO_2_ plus air.

At the end of the incubation, the cells were harvested and each sample was divided into two tubes to run parallel tests for annexin I and CXCR4 surface expression. After rinsing all samples with an HBSS solution containing 1% BSA, the cells were incubated for 45 min on a shaker with anti-annexin I or anti-CXCR4 antibodies (1:200) on ice; the negative control was incubated with an isotype-specific control antibody.

The cells were then washed twice with HBSS/BSA buffer before exposure to the Alexa Fluor® 568-conjugated secondary antibody. The excess of unbound antibody was washed away, and all samples were incubated for 15 min in the presence of annexin V-Fluos. The cells were then rinsed and resuspended in HBSS/BSA buffer and were ready for analysis in the BD FACS Canto II flow cytometry system. Electronic gates were set on annexin V-negative cells and CXCR4 or annexin I-positive cells. Data from 10,000 cells/sample were analyzed using dedicated software (Becton, Dickinson and Company). The percentage of CXCR4 or annexin I-positive cells was calculated [22].

### Western blotting

Human eosinophils were centrifuged and resuspended in 100 µl of Cytoplasmatic Extraction Reagent (CER) I buffer (included in the Nuclear and Cytoplasmatic Extraction Reagents kit). After 10 min incubation on ice, 5.5 µl of CER II buffer was added and the suspension was resuspended by vortexing, incubated on ice for 1 min, and resuspended. The cytoplasmic fraction was separated by centrifugation at 16,000× g for 5 min. The protein content was quantified using a BCA protein assay (Pierce). The proteins of the cytoplasmic extract (50 µg) were denatured at 95 °C for 3 min, then loaded and separated by 15% sodium dodecyl sulfate-PAGE. MagicMark^TM^ XP Western Standard as a molecular weight standard was used.

Proteins were transferred to Protran^TM^ nitrocellulose membranes, which were blocked with 5% nonfat milk in Tris buffered saline (10 mM Tris-HCl, pH 8, containing 150 mM NaCl) plus 0.1% Tween-20 for 1 h at room temperature (25 °C). The blots were probed overnight at 4 °C in Tris buffered saline containing 0.1% Tween-20, 5% nonfat milk, and antibodies with dilutions of 1:1,000 for caspase-3 monoclonal antibody or 1:5,000 for β-actin antibody (used as a loading control for cytoplasmic cell lysates). The former detects endogenous levels of procaspase-3 (around 32 kDa; p32) and its large subunit cleavage product of approximately 17 kDa (p17) [23]. The membranes were incubated with peroxidase-conjugated secondary antibodies at a dilution of 1:8,000. Blots were finally developed with SuperSignal West Pico chemiluminescent substrate for 5 min. The substrate was prepared by mixing (1:1) the SuperSignal West Pico Stable Peroxidase Solution and the SuperSignal West Pico Luminol/Enhancer Solution. After drainage of the solutions, chemiluminescence was acquired using a luminescent image analyzer LAS-3000 (Fujifilm, Tokyo, Japan).

### Eosinophil migration assay

The migration of human eosinophils was assayed using Transwell^TM^ inserts (pore size 5 μm) and 24-well culture plates (Corning Costar, Cambridge, MA). Briefly, the cells (2×10^5^) were suspended in 0.2 ml RPMI-1640 medium containing 0.1% BSA, treated for 2 h with mapracorat or dexamethasone (0.001–10 μM), and transferred to the upper compartment of the transwell insert. PAF (200 μl of a 10^−6^ M solution) was added in the lower compartment of the transwell insert. Vehicle-treated (control) cells contained the same concentration of ethanol used to dissolve PAF and were further diluted in RPMI 1640 containing 0.1% BSA. After 2 h incubation in an atmosphere of 95% air and 5% CO_2_, the number of cells that had migrated from the upper to the lower compartment was counted using a hemocytometer. The calculated half maximal inhibitory concentration (IC_50_) indicates the concentration of mapracorat or dexamethasone causing 50% inhibition of the maximal number of cells that had migrated in comparison to control cells.

### Cytokine and chemokine assays

Human eosinophils or HMC-1 cells (5×10^5^/ml) were suspended in RPMI 1640 containing 0.1% FBS, plated onto 24-well tissue culture plates, and pre-incubated in duplicate with dexamethasone or mapracorat for 45 min before adding ionomycin (2 μM). After 18 h stimulation at 37 °C in a 5% CO_2_ atmosphere, IL-8 was measured in supernatants obtained from eosinophils with a commercial enzyme-linked immunosorbent assay (ELISA) kit from R&D Systems. The threshold sensitivity was 5 pg/ml and the inter- and intra-assay variations were less than 5%. The supernatants obtained from HMC-1 cells were aliquoted in duplicates for IL-6, IL-8, chemokine (C-C motif) ligand 5 (CCL5)/regulated upon activation, normal T-cell expressed and secreted (RANTES), and tumor necrosis factor-α (TNF-α) analysis using high-throughput multiplex Luminex technology (Luminex 200 System; Luminex, Austin, TX) [9] and Beadview software version 1.0 (Upstate Cell Signaling Solutions, Temecula, CA). Standard curves for known concentrations of recombinant human cytokines were used to convert median fluorescence intensities to cytokine concentrations in pg/ml. Only the linear portions of the standard curves were used to quantify cytokine concentrations, and in instances where the fluorescence reading exceeded the linear range of the standard curve, an appropriate dilution was performed to ensure that the concentration was in the linear portion of the curve.

The calculated IC_50_ indicates the concentration of mapracorat or dexamethasone causing 50% inhibition of the maximal cytokine or chemokine release detected in control cells.

### Active anaphylaxis in the guinea pig

Male Dunkin-Hartley guinea pigs were actively immunized by intraperitoneal (i.p.) injection of 200 μg OVA in 2 ml saline with 40 mg aluminum hydroxide (positive control) or saline alone (negative control) [24]. This immunization procedure was repeated after one week. Three weeks after the first immunization, mapracorat and dexamethasone (0.4%, weight/volume) eye drops were instilled into the conjunctival sac (30 µl/eye) of the treated guinea pigs, and 45 min later the animals were challenged with 30 ml/eye of saline solution, containing 100 mg/ml OVA, instilled into the conjunctival sac. Negative controls received saline alone. Conjunctival clinical symptoms were rated blind on both eyes using the following scale: 0, no symptoms; 1, slight conjunctival redness with or without tears; 2, mild conjunctival redness with tears and mild chemosis; 3, mild conjunctival redness with tears and moderate chemosis; 4, severe conjunctival redness with tears and partial lid eversion; 5, severe conjunctival redness with tears and lids more than half closed. The animals were euthanized 24 h after OVA challenge by i.p. injection of Tanax^®^ (3 ml/kg; Hoechst AG, Frankfurt-am-Main, Germany), and the conjunctivas were carefully excised and each divided into two samples for subsequent investigations. One sample was fixed in 10% buffered paraformaldehyde solution and paraffin embedded; slides, 6 μm thick, were stained with Luna’s eosinophil stain to determine eosinophil accumulation and distribution. To perform Luna’s eosinophil stain, slides were desiccated in xylene, stained with hematoxylin-Biebrich scarlet solution, differentiated in 1% acid alcohol, and subsequently stained with lithium carbonate. Eosinophil granules stain red-orange [25]. The number of eosinophils in each field was counted under light microscopy (500× magnification). In the other sample eosinophil peroxidase activity was measured.

### Eosinophil peroxidase assay

Eosinophil peroxidase was assayed in conjunctival samples obtained as described above. The tissues were washed twice with ice-cold PBS, weighed, and homogenized with 50 mM Tris-HCl buffer (pH 8.0) using a Potter–Elvejehm glass/teflon homogenizer (Wheaton, Millville, NJ) on ice. After addition of 350 μl of 50 mM Tris-HCl buffer and 150 μl of 0.1% Triton X-100, the homogenates were placed in an ice bath for 1 h. The substrate solution (400 μl of 50 mM Tris-HCl buffer containing 0.1% Triton X-100, 1 mM *o*-phenylenediamine, and 0.5 mM hydrogen peroxide) was added to 200 μl of the sample and incubated at 37 °C for 10 min. The reaction was stopped with 200 μl of 4 M H_2_SO_4_. Absorbance was measured using a spectrophotometer (JASCO V-530, Jasco, Great Dunmow, Essex, UK) at 490 nm. A standard curve was plotted with different concentrations of peroxidase diluted in 50 mM Tris-HCl buffer (pH 6.0) containing 1 mM *o*-phenylenediamine and 0.5 mM hydrogen peroxide. Eosinophil peroxidase activity was measured adopting a method based on the oxidation of *o*-phenylenediamine by eosinophil peroxidase in the presence of hydrogen peroxide. One unit corresponds to 1 mmole of hydrogen peroxide decomposed for 10 min, and the results were expressed as eosinophil peroxidase levels (mU of enzyme/mg wet tissue) [24].

### Data analysis

All data were expressed as mean±standard error of the mean (SEM) for the number of experiments indicated. Statistical comparisons were made, as required, by the Student *t* test, ANOVA, and post-hoc Newman–Keuls test or two-way ANOVA with Bonferroni post test; differences of p<0.05 were considered significant. For the clinical score, each group comprised five animals. Nonparametric analysis of the scores assigned to the conjunctival symptoms was done using the Friedman test followed by Dunn's post-hoc comparison. IC_50_ or half maximal effective concentration (EC_50_) values and associated 95% confidence limits (CL) correspond to the molar drug concentration producing 50% of its own maximal effect and were generated by sigmoidal nonlinear curve fitting of the concentration-response data performed by Prism through a non-weighted iterative process (Prism version 4.0, GraphPad Software, Inc., San Diego, CA).

## Results

### Effect of mapracorat on spontaneous eosinophil apoptosis

**Figure 1:**
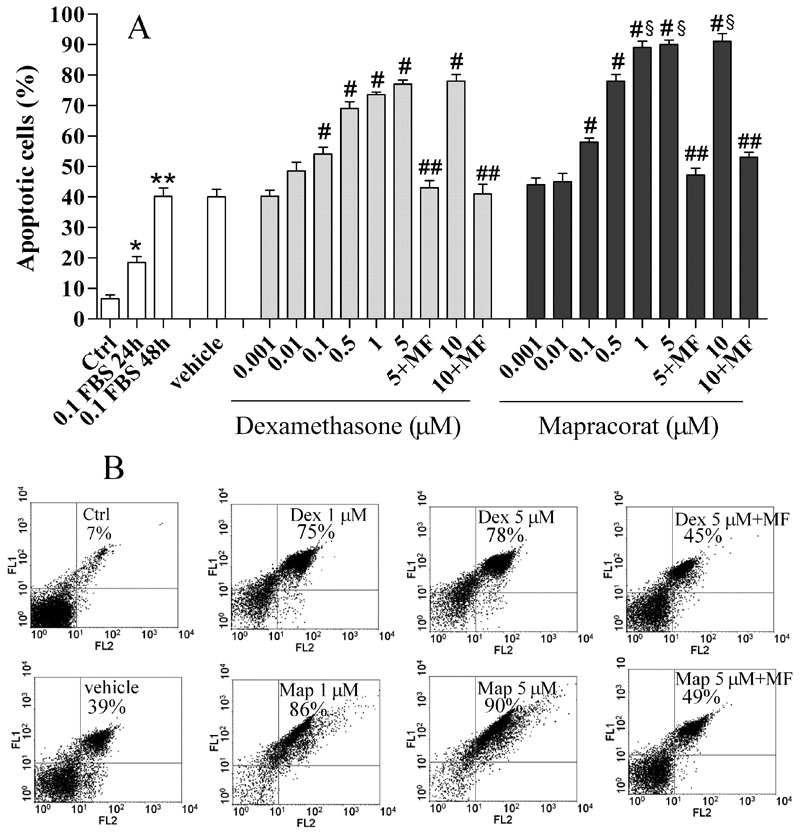
Effects of mapracorat and dexamethasone on spontaneous eosinophil apoptosis. **A**: Peripheral human blood eosinophils cultured up to 48 h in 0.1% fetal bovine serum (FBS) and in the absence of granulocyte-macrophage colony stimulating factor and interleukin-5 show time-dependent apoptosis. Mapracorat and dexamethasone (0.001–10 μM) or their vehicle were added for 48 h. Control cells (Ctrl) were cultured in RPMI+10% FBS. Mifepristone (10 μM) was co-incubated with mapracorat or dexamethasone (5 or 10 μM). Apoptosis was determined by flow cytometry, evaluating the cell’s ability to bind annexin V and exclude propidium iodide as described under Methods. Results are expressed as percentages of apoptotic cells. Data are presented as mean±standard error of the mean from six experiments performed in triplicate using different eosinophil cell cultures. *Versus controls; p value (p)<0.05. **Versus controls; p<0.01. ^#^Versus vehicle; p<0.01. ^##^Versus mapracorat or dexamethasone 5 or 10 μM; p<0.01. ^§^Versus dexamethasone at the same concentration; p<0.01. **B**: A representative experiment showing total percentage of apoptotic eosinophils (annexin V^+^/propidium iodide- and annexin V^+^/propidium iodide+ cells). Abbreviations: Ctrl represents controls; 0.1 FBS represents 0.1% fetal bovine serum; MF represents mifepristone; Dex represents dexamethasone; Map represents mapracorat.

Peripheral human blood eosinophils cultured for up to 48 h with 0.1% FBS and in the absence of prosurvival cytokines showed time-dependent spontaneous apoptosis, determined by flow cytometry to evaluate their ability to bind annexin V and exclude PI (Figure 1). Exposure of eosinophils cultured in 0.1% FBS and in the absence of prosurvival cytokines to mapracorat or to the reference glucocorticoid dexamethasone (0.001–10 μM) for 48 h enhanced constitutive eosinophil apoptosis in a concentration-dependent manner. Mapracorat was equally potent as dexamethasone but displayed a higher efficacy. IC_50_ values were mapracorat 0.142 μM (95% CL 0.021–0.941 μM) and dexamethasone 0.142 μM (95% CL 0.023–0.884 μM). Mapracorat did not cause any significant change of the concentration-response curve in comparison to dexamethasone (two-way ANOVA with Bonferroni post test); interestingly it showed higher efficacy than dexametasone (mean±SEM E*_max_*=91±2.62 versus 78±2.23; n=6; p<0.01; Student *t* test).

In eosinophils cultured with the vehicle, apoptosis was similar to cells cultured in 0.1% FBS for 48 h (Figure 1A). In eosinophils cultured in 0.1% FBS and exposed for 24 h to mapracorat and dexamethasone (0.1, 1.0, and 10 μM), a lower but significant apoptosis was observed in comparison to eosinophils cultured with the vehicle (data not shown).

Apoptosis induced by mapracorat was confirmed by the characteristic morphologic features on light microscopy reported for glucocorticoids [23], such as cell shrinkage and intense chromatin condensation (data not shown).

To confirm whether eosinophil apoptosis is induced by mapracorat through the glucocorticoid receptor, we investigated the effect of mifepristone (10 μM) [26]. This glucocorticoid receptor antagonist prevented apoptosis induced by 5 and 10 μM mapracorat or dexamethasone in human eosinophils (Figure 1).

**Figure 2:**
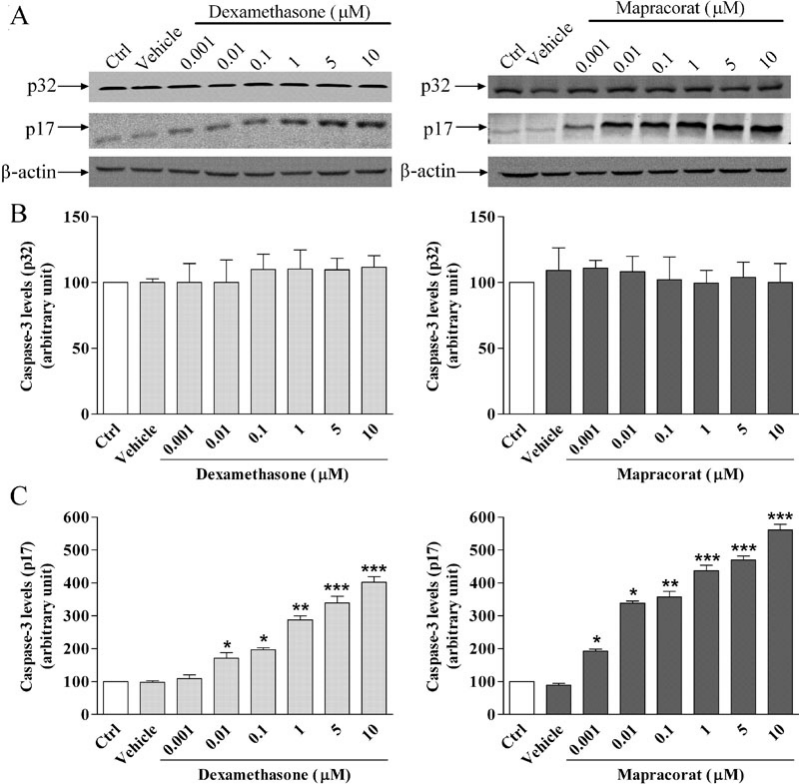
Mapracorat and dexamethasone induce caspase-3 activation in human eosinophils. Control cells were cultured for 24 h in 0.1% fetal bovine serum and in the absence of granulocyte-macrophage colony stimulating factor and interleukin-5 and were treated with mapracorat or dexamethasone (0.001–10 μM). Alternatively, eosinophils were exposed to the vehicle alone. **A**: A representative western blot, repeated at least six times using different eosinophil cell cultures, with similar results, showing the bands of apparent molecular weights of caspase-3 of approximately 32 kDa and 17 kDa and beta-actin of approximately 42 kDa. **B**: Densitometric analysis of the bands of caspase-3 fragment of approximately 32 kDa. **C**: Densitometric analysis of the bands of caspase-3 fragment of approximately 17 kDa. The approximate molecular mass of the fragments of 32 and 17 kDa was determined by comparison with molecular mass standards. The relative optical density of each band was determined by densitometry and defined by normalization of the bands of capsase-3 32 kDa or 17 kDa to the β-actin band. A total of 50 μg of protein extract was loaded and separated in a polyacrylamide gel, as described under “Methods..” Data are presented as mean±standard, n=6. *Versus controls; p value (p)<0.05. **Versus controls; p<0.01. ***Versus controls; p<0.001. Abbreviations: Ctrl represents controls; p32 represents apparent molecular weight of caspase-3 of approximately 32 kDa; p17 represents apparent molecular weight of caspase-3 of approximately 17 kDa.

To determine whether caspases were activated during these processes, caspase-3 activation during mapracorat- or dexamethasone-induced human eosinophil cell apoptosis was investigated by western blotting. In agreement with data reported in Figure 1A, human eosinophils cultured for 24 h in 0.1% FBS and in the absence of prosurvival GM-CSF and IL-5 constitutively expressed the inactive form of procaspase-3 (p32) and lower levels of its active subunit p17 (Figure 2). There was a marked concentration-dependent increase of the p17 subunit in cells exposed for 24 h to mapracorat or dexamethasone (0.001–10 μM; Figure 2). Increase of the p17subunit in cells exposed to mapracorat was higher than that observed in cells exposed to the same concentration of dexamethasone (Figure 2). We detected no changes in the amount of p32 and p17 in cells cultured for 24 h in the presence of the vehicle in comparison to control cells cultured for 24 h in 0.1% FBS and in the absence of prosurvival cytokines (control cells; Figure 2).

### Effect of mapracorat on cytokine-sustained eosinophil survival

**Figure 3:**
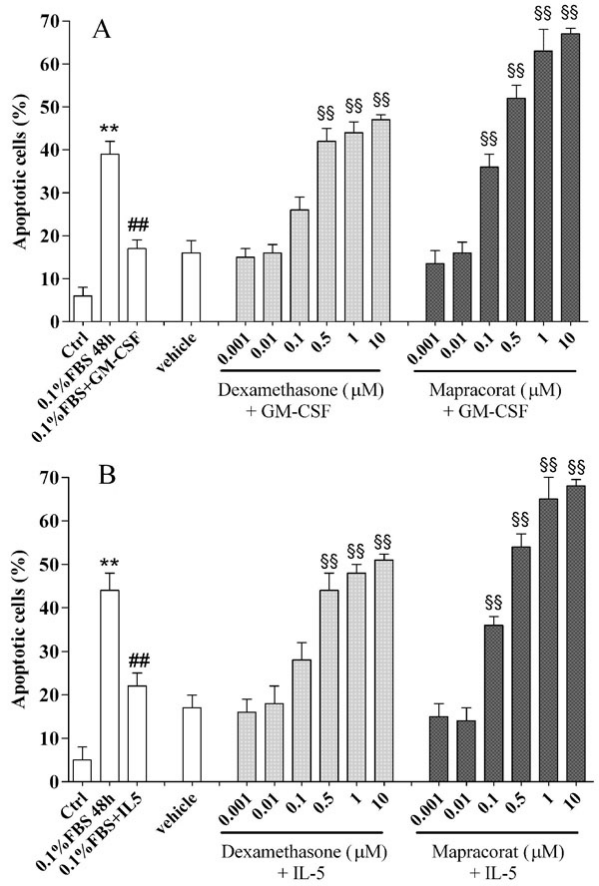
Effects of mapracorat and dexamethasone on cytokine-sustained eosinophil survival. Control eosinophils were routinely cultured in the presence of 10% fetal bovine serum or for 48 h in 0.1% fetal bovine serum and treated with granulocyte-macrophage colony stimulating factor (70 pM; **A**) or interleukin-5 (30 pM; **B**) added concomitantly with mapracorat or dexamethasone (0.001–10 μM) or their vehicle. Apoptosis was determined by flow cytometry, evaluating the cell’s ability to bind annexin V and exclude propidium iodide as described under Methods. Results are expressed as percentages of apoptotic cells. Data are presented as mean±standard error of the mean from six experiments performed in triplicate using different eosinophil cell cultures. **Versus the respective control; p value (p)<0.01. ^##^Versus 0.1% fetal bovine serum; p<0.01 ^§§^Versus 0.1% fetal bovine serum+granulocyte macrophage-colony stimulating factor or versus 0.1% fetal bovine serum + interleukin-5; p<0.01. Abbreviations: Ctrl represents controls; FBS represents fetal bovine serum; GM-CSF represents granulocyte-macrophage colony stimulating factor; IL-5 represents interleukin-5.

Prosurvival cytokines, particularly GM-CSF and IL-5, have been implicated in inhibiting eosinophil apoptosis, while glucocorticoids have been reported to reverse cytokine-sustained cell survival [22,27]. As previously described, human eosinophils cultured for 48 h in 0.1% FBS undergo significant apoptosis determined by flow cytometry to evaluate their ability to bind annexin V and exclude PI in comparison to control cells routinely maintained in medium containing 10% FBS (Figure 3). As expected, GM-CSF (70 pM) or IL-5 (30 pM) prevented eosinophil apoptosis. This effect was reversed in a concentration-dependent manner by dexamethasone or mapracorat (Figure 3A,B). Mapracorat was equally as potent as dexamethasone; inhibition of GM-CSF-induced eosinophil survival results were mapracorat IC_50_ 0.154 μM (95% CL 0.079–0.301 μM) and dexamethasone IC_50_ 0.160 μM (95% CL 0.82–0.311 μM); mapracorat did not cause any significant change in the concentration-response curve in comparison to dexamethasone (two-way ANOVA with Bonferroni post test); interestingly it showed higher efficacy than dexametasone (E*_max_*=67±2.2 versus 47±2.1; n=6; p<0.01). Inhibition of IL-5-induced eosinophil survival was mapracorat IC_50_ 0.156 μM (95% CL 0.073–0.335 μM) and dexamethasone IC_50_ 0.173 μM (95% CL 0.010–0.280 μM). Mapracorat did not cause any significant change in the concentration-response curve in comparison to dexamethasone (two-way ANOVA with Bonferroni post test), whereas it showed higher efficacy than dexametasone (E*_max_*=68±1.6 versus 51±2.3; n=6; p<0.01). However, this effect is abolished when GM-CSF or IL-5 are used at higher concentrations [27]. Dexamethasone and mapracorat were not, in fact, able to reverse cytokine-sustained survival in the presence of GM-CSF 200 pM or IL-5 100 pM (data not shown).

### Effect of mapracorat on eosinophil migration induced by platelet-activating factor

PAF (10^−6^ M) added in the lower compartment of transwell inserts induced a significant increase of eosinophil migration (mean±SEM 1,830±38 cells; n=6) over controls (172±34 cells; n=6, p<0.01). Mapracorat and dexamethasone (0.001–10 μM) caused a concentration-dependent reduction of eosinophil migration from the upper to the lower compartment of the transwell insert. Mapracorat was equally as potent as dexamethasone; inhibition of PAF-induced eosinophil migration results were mapracorat IC_50_ 0.114 μM (95% CL 0.025–0.313 μM) and dexamethasone IC_50_ 0.148 μM (95% CL 0.035–0.402 μM); mapracorat did not cause any significant change of the concentration-response curve in comparison to dexamethasone (two-way ANOVA with Bonferroni post test); interestingly it showed higher efficacy than dexamethasone (E*_max_*=93±4.0 versus 74±6.5; n=6; p<0.01). Mifepristone (10 μM) co-incubated with mapracorat or dexamethasone (5 or 10 μM) prevented eosinophil migration (data not shown).

### Mapracorat has less transactivation activity than dexamethasone

**Figure 4:**
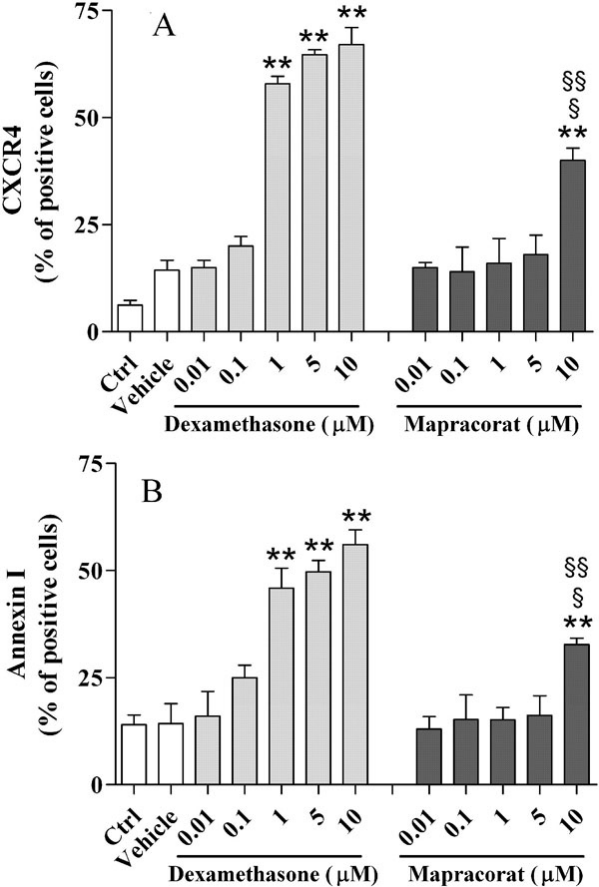
Effects of mapracorat and dexamethasone on CXCR4 receptor and annexin I surface expression in human eosinophils. Eosinophils were routinely cultured in 10% fetal bovine serum containing prosurvival granulocyte-macrophage colony stimulating factor and interleukin-5 (controls); alternatively, eosinophils were maintained for 48 h in 0.1% fetal bovine serum lacking granulocyte-macrophage colony stimulating factor and interleukin-5 and were treated with mapracorat or dexamentasone (0.01–10 μM) or their vehicle. C-X-C-chemokine receptor 4 (CXCR4) or annexin 1 expression was evaluated by flow cytometry analysis as described under Methods. CXCR4 receptor expression (**A**) and annexin I expression (**B**) are presented as percentages of positive cells and calculated as described under Methods. Data are presented as mean±standard error of the mean from six experiments performed in triplicate using different eosinophil cell cultures. **Versus vehicle; p (value) p<0.01. ^§^Versus dexamethasone 1 μM; p<0.05. ^§§^Versus dexamethasone 5 and 10 μM; p<0.01. Abbreviations: Ctrl represents controls.

Activated glucocorticoid receptors bind recognition sites in the promoters of certain genes to activate their transcription; this is known as transactivation. The CXCR4 receptor and annexin I can be considered markers of glucocorticoid-induced transactivation [22]. To determine whether mapracorat maintains transactivation on binding to the glucocorticoid receptor, we used flow cytometry to determine the induction of CXCR4 receptor and annexin I in eosinophil cells exposed for 24 h to mapracorat (0.01–10 μM) in comparison to the positive effect elicited by dexamethasone (0.01–10 μM). As reported in Figure 4A, dexamethasone (1, 5, and 10 μM) induced a significant concentration-dependent increase of the CXCR4 receptor expression; conversely, mapracorat up to 5 μM did not change CXCR4 receptor expression in comparison to vehicle-treated or control eosinophils. However, 10 μM mapracorat partially increased this receptor on the cell surface; this elevation was significantly lower than that induced by 1, 5, and 10 μM dexamethasone. Results were similar for annexin I, which is the other marker of glucocorticoid-induced transactivation investigated here (Figure 4B). These data indicate that mapracorat is less potent than dexamethasone in activating transactivation mechanisms regulated by glucocorticoid agents.

The upregulating effect of 1, 5, and 10 μM dexamethasone or 10 μM mapracorat on CXCR4 or annexin I expression cannot be explained by its apoptosis-inducing activity. Treated eosinophil cells were double stained with annexin V and anti-CXCR4 or anti-annexin I and their expression was detected in cells stained negatively with annexin V.

### Effect of mapracorat on cytokine secretion

Glucocorticoids inhibit cytokine production and secretion in immune cells [11]. This has been called transrepression and contributes to their anti-inflammatory activity [7,15]. In view of the substantial apoptosis caused by mapracorat in peripheral blood eosinophils, we investigated its effect on IL-8 release from ionomycin-treated eosinophils. IL-8 is produced by eosinophils [28] and is involved in eosinophil migration and survival, which are two relevant aspects in chronic allergic diseases [29]. We also investigated the compound’s action on cytokine and chemokine secretion in the human mast cell line HMC-1 as these can greatly influence eosinophil activity in inflamed ocular tissues [2].

**Figure 5:**
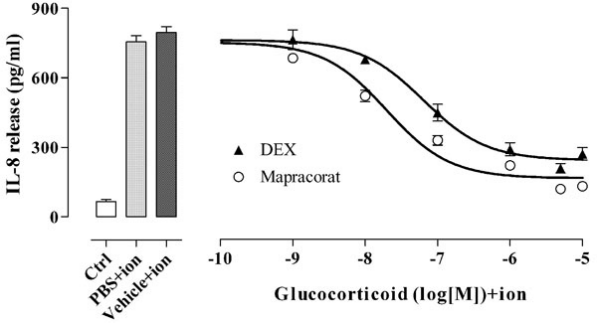
Effects of mapracorat and dexamethasone on interleukin-8 (IL-8) secretion induced by ionomycin in eosinophils. Cells (0.5x10^6^ cells/well) were suspended in cell culture medium containing 0.1% fetal bovine serum and exposed to phosphate buffered saline (PBS) or to the vehiche; alternatively, cells were treated with mapracorat or dexamethasone (0.001–10 μM); after 45 min, ionomycin (2 μM) was added. Controls were not exposed to ionomycin. IL-8 was assayed by an enzyme-linked immunosorbent assay on supernatant samples collected 18 h later, as described under Methods. Data are presented as mean±standard error of the mean from six experiments performed in triplicate using different eosinophil cell cultures (for the sake of clarity some error bars are not reported). Abbreviations: Ctrl, represents controls; ion represents ionomycin; DEX represents dexamethasone. Mapracorat did not cause any significant change of the concentration-response curve in comparison to dexamethasone (two-way ANOVA with Bonferroni post test).

Mapracorat and dexamethasone (0.001–10 μM) both reduced IL-8 release induced by ionomycin in eosinophils cells in a concentration-related manner (Figure 5). Mapracorat displayed potency similar to dexamethasone (mapracorat IC_50_ 0.020 μM, 95% CL 0.013– 0.132 μM; dexamethasone IC_50_ 0.064 μM, 95% CL 0.0037–0.109 μM) and did not cause any significant change of the concentration-response curve in comparison to dexamethasone (two-way ANOVA with Bonferroni post test). Similarly, both antagonized the release induced by ionomycin of the following cytokines from HMC-1 cells: IL-6, IL-8, CCL5/RANTES, and TNF-α (IC_50_ results are reported in Table 1). Mapracorat was equally as potent as dexamethasone in inhibiting ionomycin-induced secretion of IL-6, IL-8, CCL5/RANTES, and TNF-α.

**Table 1:** Inhibitory effect of mapracorat and dexamethasone on the release of interleukin-6, interleukin-8, TNF-α, and CCL5/RANTES induced by ionomycin in human HMC-1 cells.

**Cytokine or chemokine assayed**	**Mapracorat IC_50_ (μM)**	**Dexamethasone IC_50_ (μM)**
Interleukin-6	0.141^a^ (0.053–0.320)^b^	0.093 (0.045–0.165)
Interleukin-8	0.063 (0.039–0.164)	0.060 (0.025–0.117)
TNF-α	0.167 (0.041–0.182)	0.144 (0.056–0.298)
CCL5/RANTES	0.086 (0.044–0.398)	0.116 (0.061–0.597)

The effect of mapracorat on secretion of the reported cytokines and chemokines, 18 h after ionomycin (2 μM) stimulation of HMC-1 cells, was determined in comparison to dexamenthasone. Compounds (0.001–10 μM) were added 45 min before ionomycin. Values were calculated by analyzing at least three separated experiments performed in duplicate, where concentration-response curves were measured. ^a^For each cytokine or chemokine assayed, mapracorat did not cause any significant change of the concentration-response curve in comparison to dexamethasone (two-way ANOVA with Bonferroni post-test). ^b^95% Confidence limits.

### Effect of mapracorat on conjunctival symptoms and conjunctival eosinophil recruitment in ovalbumin-sensitized guinea pigs

**Figure 6:**
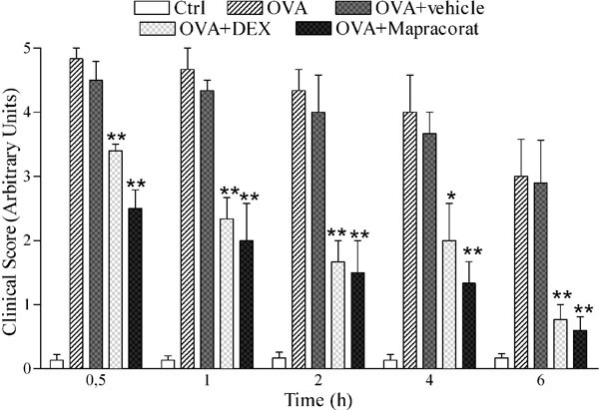
Effects of mapracorat and dexamethasone on conjunctival symptoms induced by ovalbumin in guinea pigs. Mapracorat and dexamethasone eye drops were administered to guinea pigs actively immunized by i.p. injection of ovalbumin (OVA) and 2 weeks later challenged with OVA (30 µl of 2.5% solution) instilled into both eyes; 45 min before this challenge mapracorat (0.4%), dexamethasone (0.4%), or the vehicle (phosphate buffered saline) were instilled into both eyes (30 µl/eye). Controls received the vehicle alone and were not treated with OVA. Each group comprised five guinea pigs, and the score was based on changes before and 1,2, 4, and 6 h after challenge for the symptoms of itching, swelling, redness, and lid eversion as described under Methods. Data are presented as mean±standard error of the mean, n=10 (both eyes were evaluated). *Versus OVA or OVA+vehicle; p (value) p<0.05. **Versus OVA or OVA+vehicle; p<0.01. Abbreviations: Ctrl represents controls; OVA represents ovalbumin; DEX represents dexamethasone.

Guinea pigs were actively immunized by i.p. injection of OVA and 2 weeks later were challenged with OVA instilled into the conjunctival sac. One hour after challenge, during the early phase ocular reaction, swelling of the eyelids and chemosis were more marked in treated animals than controls, but the difference was significantly reduced by 0.4% mapracorat or dexamethasone eye drops given before treatment (30 µl/eye 45 min before OVA; Figure 6). During the late phase of allergic conjunctivitis, 6 h after challenge, there was still a significant reduction in the severity of conjunctival symptoms in treated guinea pigs with both compounds.

**Figure 7:**
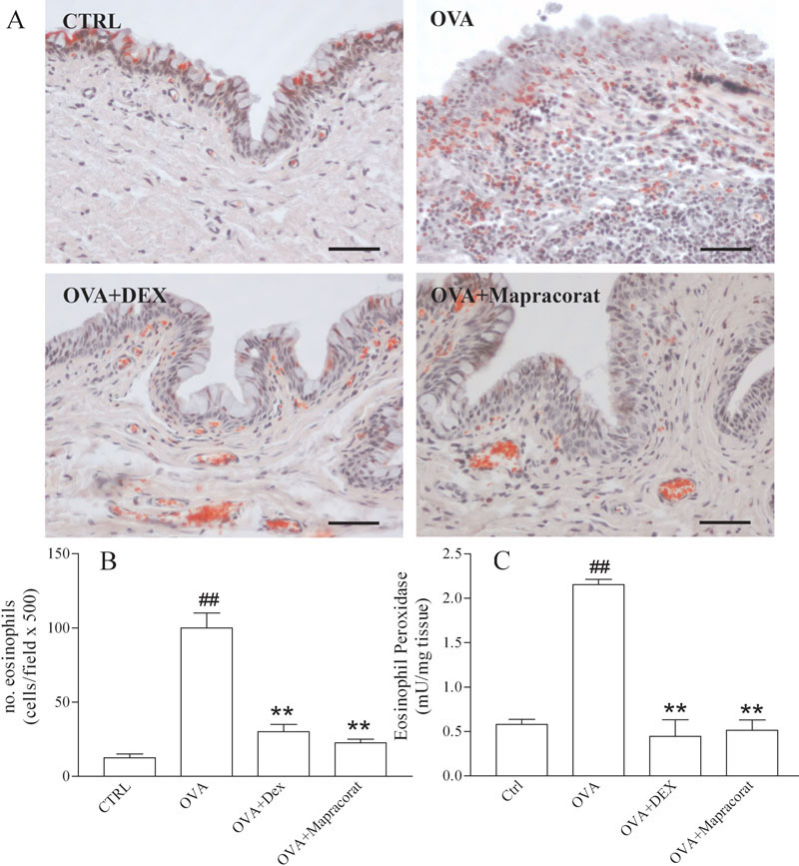
Effects of mapracorat and dexamethasone eye drops on eosinophil infiltration induced by ovalbumin in the guinea pig conjunctiva (details are reported in the legend of Figure 6). **A**: Photomicrographs of the conjunctiva 24 h after topical challenge with ovalbumin. Substantial eosinophil infiltration is observed in ovalbumin-treated guinea pigs in comparison to negative controls treated with saline alone and not challenged with ovalbumin. In guinea pigs treated with mapracorat or dexamethasone eye drops and 45 min later with ovalbumin, there was much less eosinophil infiltration than in conjunctiva of guinea pigs treated with ovalbumin alone. **B**: Effects of mapracorat and dexamethasone eye drops on conjunctival eosinophil infiltration 24 h after topical challenge with ovalbumin. The eosinophils in each field were counted 24 h after antigen exposure. Controls received saline alone and were not challenged with ovalbumin. **C**: Effects of mapracorat or dexamethasone eye drops on conjunctival eosinophil peroxidase levels 24 h after topical challenge with ovalbumin. Controls received saline alone and were not challenged with ovalbumin. **Versus ovalbumin; p value (p)<0.01. ^##^Versus controls; p<0.01. The original pictures were taken at 500x magnification. The scale bar represents 50 μm. Abbreviations: CTRL represents controls; OVA represents ovalbumin; DEX represents dexamethasone.

The guinea pigs were euthanized by intraperitoneal injection of 3 ml/kg of Tanax® 24 h later and histological analysis showed numerous eosinophils infiltrating the conjunctiva. The infiltration was much less marked in mapracorat- or dexamethasone-treated guinea pigs than in OVA-treated animals (Figure 7A,B). Similarly, eosinophil peroxidase activity, taken as an indicator of eosinophil infiltration, increased 24 h after antigen challenge in OVA-treated guinea pigs, whereas there was a noteworthy reduction in mapracorat or dexamethasone-treated animals (Figure 7C).

## Discussion

Schäcke et al. [15] recently described the pharmacological profile of the novel dissociated glucocorticoid ligand mapracorat, which was proposed for topical application to treat skin disorders. This compound binds with high affinity and selectivity to the human glucocorticoid receptor, inhibits in vitro cytokine secretion from peripheral blood mononuclear cells, blocks T-cell proliferation and, when topically administered in vivo in two models of contact dermatitis, has strong anti-inflammatory activity.

This study investigated the potential anti-allergic activity of topical mapracorat in the eye and its effects in vitro on eosinophil functions and cytokine secretion. We focused on eosinophils since these cells mediate unique cytotoxic and inflammatory effects by the generation, storage, and release of their granule proteins and the production of cytokines, growth factors, reactive oxygen species, and pro-inflammatory lipid mediators [4]. Their recruitment and activation are regarded as crucial to the development of allergic disorders, including conjunctivitis [1]. Besides selective migration, longer cell survival and decreased apoptosis are relevant to tissue-specific accumulation of these inflammatory cells [4].

Glucocorticoids are the most effective anti-inflammatory drugs used to treat eosinophil disorders as they can prevent eosinophil accumulation and activation and induce eosinophil apoptosis [26,30,31]. We found that mapracorat, binding to the glucocorticoid receptor, displayed potency similar to that of dexamethasone and was more effective in increasing spontaneous eosinophil apoptosis and counteracting cytokine-sustained eosinophil survival; interestingly the difference between the two drugs is maintained when their concentration reaches 10 μM. This was clear after 48 h of treatment in peripheral human blood eosinophils. Furthermore, we proved that mapracorat caused a concentration-dependent inhibition of PAF-induced eosinophil migration. Involvement of the glucocorticoid receptor was suggested by the effect of the glucocorticoid receptor antagonist mifepristone [26] as it prevented mapracorat- or dexamethasone-induced apoptosis. Taken together, these results suggest that the inhibitory effect of mapracorat on eosinophil accumulation observed in vivo at the conjunctival level may involve various mechanisms, including eosinophil apoptosis and their recruitment and activation or release of cytokines and chemokines [32]. The contribution of glucocorticoids to eosinophil apoptosis in allergic diseases in vivo remains to be further investigated [33].

Although the death signal that triggers the apoptotic program can originate from different sources, the signaling pathways ultimately lead to the activation of a family of cysteine proteases known as caspases [34]. We showed that mapracorat, like dexamethasone, activates caspase-3 by interacting with the glucocorticoid receptor. Its apoptotic effect on eosinophils might contribute in vivo to their rapid removal by phagocytes to prevent their accumulation and the release of cytotoxic proteins [31].

Schäcke et al. [15] reported that mapracorat, unlike classical glucocorticoids, does not induce apoptosis in the murine thymocyte line S49. This difference calls for further exploration employing human thymocytes. However, according to Druilhe et al. [4] glucocorticoids may activate different signaling pathways in these cells, and the marked differences in the kinetics of glucocorticoid-induced death in thymocytes (2–6 h) and eosinophils (24–48 h) must be borne in mind.

Besides differences in species (human eosinophils versus murine thymocytes), one possible explanation of the mapracorat-induced eosinophil apoptosis is that its repressor activity, which requires longer exposure, predominates in the control of eosinophil apoptosis. This is borne out by present data and previous studies [9,15] indicating that mapracorat has a preference for repression mechanisms rather than activation at a transcriptional level. This unique profile might be due to its binding to the glucocorticoid receptor, which leads to a change in receptor conformation. This could induce different binding with other co-factors and/or with glucocorticoid recognition elements residing in the promoter of target genes. Helmberg et al. [35] suggested that interference with pro-inflammatory signaling through transrepressional activity is an important mechanism of glucocorticoid-induced apoptosis. However, induction of the expression of pro-apoptotic agents [31] or a potential effect elicited by mapracorat on intracellular signaling involved in this process [36] cannot be ruled out.

The present results for mapracorat cannot be a consequence of its degradation as Pfeffer et al. [17] have shown that this compound is stable under conditions similar to those adopted in the in vitro models used in the present study.

We confirmed that mapracorat has reduced transactivation activity as it was partially effective only at the highest concentration (10 μM) in inducing the expression of the CXCR4 receptor and of annexin I on the eosinophil cell surface. Conversely, the reference compound dexamethasone was active at a concentration ten times lower (1 μM).

CXCR4 is a constitutive chemokine receptor that is widely expressed on leukocytes and enhances the active retention of highly differentiated primed T cells at sites of chronic inflammation [37]. These observations are interesting because in vivo studies have indicated that topical glucocorticoids may potently upregulate CXCR4 expression on primed T lymphocytes in the aqueous humor of patients with uveitis [38]. In eosinophils, the expression of CXCR4 is functional; a specific ligand for CXCR4, stromal cell-derived factor 1α (SDF-1α), can elicit strong migration, comparable with that of eotaxin [39]. Therefore, the finding that mapracorat is a weaker activator than dexamethasone of CXCR4 expression in eosinophils could be favorable for anti-allergic activity. However, in vivo evidence of the role of glucocorticoids in CXCR4 expression in eosinophils is still lacking, and further investigations are necessary to clarify this receptor’s intriguing role in eosinophil recruitment.

As regards the reduced transactivational activity of mapracorat-induced annexin I expression, this might negatively influence its anti-allergic properties [40]. Annexin I on the eosinophil surface is upregulated by glucocorticoids and prevents integrin adhesion, which is essential to cell migration [41]. Again, in vivo studies aimed to evaluate the effect of mapracorat on annexin I expression are needed. However, this effect does not affect the anti-allergic activity of mapracorat as we found it had potent anti-allergic activity in OVA-sensitized guinea pigs. Furthermore, a recent study has reported that mapracorat acts as a partial glucocorticoid receptor agonist in increasing a moderate elevation of myocilin expression in trabecular meshwork cells—an effect that may be due to its peculiar regulation of transactivation mechanisms [17].

Gene repression modulated by mapracorat can contribute indirectly to eosinophil apoptosis and/or activation by inhibiting cytokine and chemokine production and secretion by the eosinophils and mast cells [42]. This agent caused concentration-related inhibition of IL-8 release from eosinophils and the release of IL-6, IL-8, CCL5/RANTES, and TNF-α from HMC-1 human mast cells. In agreement with our findings, Zhang et al. [9] and Cavet et al. [19] have reported that mapracorat may act, at an ocular level, as a potent anti-inflammatory agent as it blocks the release of various cytokines and chemokines in various primary human ocular cells with similar activity and potency as dexamethasone. These effects help to explain the potent anti-allergic effect of mapracorat in reducing the conjunctival symptoms and conjunctival eosinophil accumulation in OVA-sensitized guinea pigs.

This novel compound behaves as the full glucocorticoid receptor agonist dexamethasone and has beneficial effects on early and late-phase inflammatory changes induced by the allergen-specific conjunctival challenge. Histamine and eicosanoids are responsible for the typical early phase response [3]. However, mast cells also contribute to the synthesis and release of cytokines, chemokines, and growth factors, triggering a cascade of inflammatory events on the surface of epithelial and endothelial cells that leads to the late-phase response, with recruitment of eosinophils and neutrophils [3]. Therefore mapracorat, as suggested by Zhang et al. [9] and Cavet et al. [19], may act on different cell types involved in the complex inflammatory response in the eye by influencing the production of pro-inflammatory cytokines and chemokines as well as inducing eosinophil apoptosis. These effects appear to be predominantly regulated by the transrepressional arm of glucocorticoid action [7].

In terms of separating transactivation from transrepression, it is clear that many genes regulated through transactivation are not represented in current screening assays. Thus, the dissociation actually shown by these novel compounds obviously needs further investigation [8,14]. Several glucocorticoid-inducible genes contribute to their anti-inflammatory action, and the loss of any transactivational properties might reduce this [11]. Therefore, it is essential to verify the anti-inflammatory activity of these novel glucocorticoid receptor ligands in vivo in models where both favorable and unfavorable transactivation and transrepression events occur. Finally, as reported by Newton and Holden [14], it would be better to search for “differential” compounds that show the most favorable functional profiles rather than searching for glucocorticoid ligands that distinguish transrepression and transactivation.

In conclusion, mapracorat seems to be a promising candidate for the topical treatment of allergic eye disorders. It easily appears to reach conjunctival cells and vessels when administered topically [43], and some of its cellular targets may contribute to eosinophil apoptosis and/or to preventing their recruitment and activation and to inhibiting the release of cytokines and chemokines. Future studies should further explore its safety profile and better define its pharmacodynamic profile.

## References

[1] Leonardi A, Motterle L, Bortolotti M (2008). Allergy and the eye.. Clin Exp Immunol.

[2] Miyazaki D, Tominaga T, Yakura K, Kuo CH, Komatsu N, Inoue Y, Ono SJ (2008). Conjunctival mast cell as a mediator of eosinophilic response in ocular allergy.. Mol Vis.

[3] Ono SJ, Abelson MB (2005). Allergic conjunctivitis: update on pathophysiology and prospects for future treatment.. J Allergy Clin Immunol.

[4] Druilhe A, Letuve S, Pretolani M (2003). Glucocorticoid-induced apoptosis in human eosinophils: mechanisms of action.. Apoptosis.

[5] Frauman AG (1996). An overview of the adverse reactions to adrenal corticosteroids.. Adverse Drug React Toxicol Rev.

[6] Carnahan MC, Goldstein DA (2000). Ocular complications of topical, peri-ocular, and systemic corticosteroids.. Curr Opin Ophthalmol.

[7] Biddie SC, Hager GL (2009). Glucocorticoid receptor dynamics and gene regulation.. Stress.

[8] Catley M (2007). Dissociated steroids.. ScientificWorldJournal.

[9] Zhang JZ, Cavet ME, VanderMeid KR, Salvador-Silva M, Lopez FJ, Ward KW (2009). BOL-303242-X, a novel selective glucocorticoid receptor agonist, with full anti-inflammatory properties in human ocular cells.. Mol Vis.

[10] De Bosscher K, Haegeman G (2009). Minireview: latest perspectives on antiinflammatory actions of glucocorticoids.. Mol Endocrinol.

[11] Clark AR (2007). Anti-inflammatory functions of glucocorticoid-induced genes.. Mol Cell Endocrinol.

[12] Schäcke H, Döcke WD, Asadullah K (2002). Mechanisms involved in the side effects of glucocorticoids.. Pharmacol Ther.

[13] McMaster A (2008). Ray DW Drug insight: selective agonists and antagonists of the glucocorticoid receptor.. Nat Clin Pract Endocrinol Metab.

[14] Newton R, Holden NS (2007). Separating transrepression and transactivation: a distressing divorce for the glucocorticoid receptor?. Mol Pharmacol.

[15] Schäcke H, Zollner TM, Docke WD, Rehwinkel H, Jaroch S, Skuballa W, Neuhaus R, May E, Zügel U, Asadullah K (2009). Characterization of ZK 245186, a novel, selective glucocorticoid receptor agonist for the topical treatment of inflammatory skin diseases.. Br J Pharmacol.

[16] Shafiee A, Bucolo C, Budzynski E, Ward KW, Lopez FJ (2011). In vivo ocular efficacy profile of BOL-303242-X, a novel selective glucocorticoid receptor agonist, in rabbit models of ocular disease.. Invest Ophthalmol Vis Sci.

[17] Pfeffer BA, DeWitt CA, Salvador-Silva M, Cavet ME, Lopez FJ, Ward KW (2010). Reduced myocilin expression in cultured monkey trabecular meshwork cells induced by a selective glucocorticoid receptor agonist: comparison with steroids.. Invest Ophthalmol Vis Sci.

[18] Sohn S, Hur W, Cho YR, Chung YS, Ki C-S, Kee C (2010). Little evidence for association of the glaucoma gene MYOC with open-angle glaucoma.. Br J Ophthalmol.

[19] Cavet ME, Harrington KL, Ward KW, Zhang J-Z (2010). Mapracorat, a novel selective glucocorticoid receptor agonist, inhibits hyperosmolar-induced cytokine release and MAPK pathways in human corneal epithelial cells.. Mol Vis.

[20] Nilsson G, Blom T, Kusche-Gullberg M, Kjellen L, Butterfield JH, Sundstrom C, Nilsson K, Hellman L (1994). Phenotypic characterization of the human mast-cell line HMC-1.. Scand J Immunol.

[21] Kankaanranta H, Janka-Junttila M, Ilmarinen-Salo P, Ito K, Jalonen U, Ito M, Adcock IM, Moilanen E, Zhang X (2010). Histone deacetylase inhibitors induce apoptosis in human eosinophils and neutrophils.. J Inflamm (Lond).

[22] Janka-Junttila M, Moilanen E, Hasala H, Zhang X, Adcock I, Kankaanranta H (2006). The glucocorticoid RU24858 does not distinguish between transrepression and transactivation in primary human eosinophils.. J Inflamm (Lond).

[23] Létuvé S, Druilhe A, Grandsaigne M, Aubier M, Pretolani M (2002). Critical role of mitochondria, but not caspases, during glucocorticosteroid-induced human eosinophil apoptosis.. Am J Respir Cell Mol Biol.

[24] Qasem AR, Bucolo C, Baiula M, Sparta A, Govoni P, Bedini A, Fascì D, Spampinato S (2008). Contribution of alpha4beta1 integrin to the antiallergic effect of levocabastine.. Biochem Pharmacol.

[25] Luna LG. Manual of histologic staining methods of the Armed Forces Institute of Pathology. Third Edition. New York: Mc Graw Hill Co.; 1968. p. 111–2.

[26] Zhang X, Moilanen E, Kankaanranta H (2000). Enhancement of human eosinophil apoptosis by fluticasone propionate, budesonide, and beclomethasone.. Eur J Pharmacol.

[27] Hagan JB, Kita H, Gleich GJ (1998). Inhibition of interleukin-5 mediated eosinophil viability by fluticasone 17-propionate: comparison with other glucocorticoids.. Clin Exp Allergy.

[28] Cheng G, Ueda T, Nakajima H, Nakajima A, Kinjyo S, Motojima S, Kukuda T (1998). Suppressive effects of SP-A on ionomycin-induced IL-8 production and release by eosinophils.. Int Arch Allergy Immunol.

[29] Park HS, Jung KS, Shute J, Roberts K, Holgate ST, Djukanovic R (1997). Allergen-induced release of GM-CSF and IL-8 in vitro by nasal polyp tissue from atopic subjects prolongs eosinophil survival.. Eur Respir J.

[30] Chauhan S, Leach CH, Kunz S, Bloom JW, Miesfeld RL (2003). Glucocorticoid regulation of human eosinophil gene expression.. J Steroid Biochem Mol Biol.

[31] McColl A, Michlewska S, Dransfield I, Rossi AG (2007). Effects of glucocorticoids on apoptosis and clearance of apoptotic cells.. ScientificWorldJournal.

[32] Sugimoto Y, Ogawa M, Tai N, Kamei C (2003). Inhibitory effects of glucocorticoids on rat eosinophil superoxide generation and chemotaxis.. Int Immunopharmacol.

[33] Uller L, Lloyd CM, Rydell-Törmänen K, Persson CG, Erjefält JS (2006). Effects of steroid treatment on lung CC chemokines, apoptosis and transepithelial cell clearance during development and resolution of allergic airway inflammation.. Clin Exp Allergy.

[34] Simon HU (2009). Cell death in allergic diseases.. Apoptosis.

[35] Helmberg A, Auphan N, Caelles C, Karin M (1995). Glucocorticoid-induced apoptosis of human leukemic cells is caused by the repressive function of the glucocorticoid receptor.. EMBO J.

[36] Stahn C, Buttgereit F (2008). Genomic and nongenomic effects of glucocorticoids.. Nat Clin Pract Rheumatol.

[37] Okada T, Ngo VN, Ekland EH, Forster R, Lipp M, Littman DR, Cyster JG (2002). Chemokine requirements for B cell entry to lymph nodes and Peyer's patches.. J Exp Med.

[38] Curnow SJ, Wloka K, Faint JM, Amft N, Cheung CM, Savant V, Lord J, Akbar AN, Buckley CD, Murray PI, Salmon M (2004). Topical glucocorticoid therapy directly induces up-regulation of functional CXCR4 on primed T lymphocytes in the aqueous humor of patients with uveitis.. J Immunol.

[39] Nagase H, Miyamasu M, Yamaguchi M, Fujisawa T, Kawasaki H, Ohta K, Yamamoto K, Morita Y, Hirai K (2001). Regulation of chemokine receptor expression in eosinophils.. Int Arch Allergy Immunol.

[40] Scannell M, Maderna P (2006). Lipoxins and annexin-1: resolution of inflammation and regulation of phagocytosis of apoptotic cells.. ScientificWorldJournal.

[41] Liu J, Zhu X, Myo S, Lambertino AT, Xu C, Boetticher E, Muñoz NM, Sano M, Cordoba M, Learoyd J, Meliton A, Johnson M, Leff AR (2005). Glucocorticoid-induced surface expression of annexin 1 blocks beta2-integrin adhesion of human eosinophils to intercellular adhesion molecule 1 surrogate protein.. J Allergy Clin Immunol.

[42] Blanchard C, Rothenberg ME (2009). Biology of the eosinophil.. Adv Immunol.

[43] Proksch JW, Lowe ER, Ward KW (2011). Ocular pharmacokinetics of mapracorat, a novel selective glucocorticoid receptor agonist, in rabbits and monkeys.. Drug Metab Dispos.

